# Co-morbid cannabis use disorder and chronotype are associated with mood symptom onset in people with bipolar disorder

**DOI:** 10.1016/j.jpsychires.2024.11.007

**Published:** 2024-11-04

**Authors:** Alannah Miranda, Breanna M. Holloway, William Perry, Arpi Minassian, Michael McCarthy

**Affiliations:** a Department of Psychiatry, University of California San Diego, San Diego, CA, USA; b Veterans Affairs San Diego Healthcare System, La Jolla, CA, USA

**Keywords:** Cannabis abuse, Bipolar disorder, Mania, Depression, Chronotype

## Abstract

Comorbid cannabis use disorder (CUD) is disproportionately high in people with bipolar disorder (BD) and has been associated with worsening of BD symptoms. However, many people with BD report regularly using cannabis to ameliorate symptoms, including sleep disturbances. Sleep and circadian rhythm disturbances are hallmark features of BD that often precede the onset of mood symptoms. Genetic studies indicate that circadian disruption may predispose individuals towards both problematic cannabis use and BD, rather than cannabis use directly impacting BD symptoms. To further disentangle these hypotheses, we aimed to investigate the relationship between chronotype, cannabis use disorder (CUD) and BD mood symptoms. Data from 212 participants with BD I from the Pharmacogenomics of Bipolar Disorder study dataset were analyzed for this study. Participants were stratified by those diagnosed with co-morbid CUD and BD symptom variables, including the mean number of mood episodes per year and age of mood symptom onset for both depression and mania symptoms. The Basic Language Morningness scale (BALM) was used to assess chronotype. There was no interaction between morningness levels and CUD on BD symptoms, however both lower morningness and CUD were independently associated with earlier age of mood symptom onset. However, patients who reported initiating cannabis use post mood symptom onset had an earlier mood symptom age of onset compared to those who reported initiating cannabis use prior to mood symptom onset. These findings could provide further evidence that circadian rhythm disruption could be an underlying factor that predisposes individuals toward both CUD and BD.

## 1. Introduction

Bipolar disorder (BD) is a neuropsychiatric disorder defined by recurrent mood shifts between depressive and manic/hypomanic episodes that contribute to poor psychosocial and health outcomes. In addition to mood episodes, people with BD are at a higher risk for co-morbid health outcomes which may further worsen their quality of life. Substance use disorders (SUD) are amongst the most prominent co-occurring conditions that present in people with BD. In fact, compared to other serious mental illnesses, BD has amongst the highest comorbidity of SUD(Gold et al., 2018). The high prevalence of co-morbid BD and SUD could indicate an underlying cause that contributes to an increased susceptibility towards developing both conditions. Circadian rhythm disturbances may increase susceptibility towards a co-morbid substance use disorder. Circadian rhythm disruptions are a hallmark feature of BD (Gold and Sylvia, 2016; Harvey et al., 2009) that have also been implicated as a contributing factor towards substance abuse(Hasler et al., 2014; Tamura et al., 2021). Notably, circadian rhythms disruptions in people with BD can worsen sleep and mood symptoms and adversely affect clinical course and treatment(Takaesu et al., 2018), including substance use outcomes(Serrano-Serrano et al., 2021). In particular, evening chronotype (i.e., a tendency to sleep and wake later and proxy for circadian phase delay) has been associated with a higher risk of BD as well as substance use and relapse (Berdynaj et al., 2016; Menculini et al., 2023; Santos et al., 2023; Serrano-Serrano et al., 2021).

Amongst other forms of substance use, cannabis use is one of the most prevalent in people with BD, with up to 70% reporting a lifetime history of regular cannabis use and up to 20% with a comorbid cannabis use disorder (CUD) diagnosis(Agrawal et al., 2011). Cannabis is also commonly used for its reputed sleep-promoting properties(Babson et al., 2017; Monti and Pandi-Perumal, 2022). In fact, 70% of people with BD reported using cannabis specifically to address sleep disturbances in one study(Miranda et al., 2023). However, the effects of cannabis on circadian regulation of sleep remain unclear; thus, there remain concerns that cannabis may interact with the compromised circadian system in BD and further exacerbate symptoms. Notably, cannabis use has been associated with a significantly younger age of schizophrenia onset (Veen et al., 2004) and age of onset at first psychosis treatment (Barrigón et al., 2010). Cannabis use has been associated with worse mania and psychosis symptoms(Sideli et al., 2019), an earlier age of BD onset (Lagerberg et al., 2011), and overall worse BD illness progression(Pinto et al., 2019). As such, cannabis use has been identified as a potential risk factor for the development of BD and the worsening of BD symptoms.

As an alternative hypothesis to cannabis use being a risk factor for BD or worsening of BD symptoms, genetic studies suggest that CUD and BD may instead have a shared underlying mechanism that predisposes individuals towards the development of both conditions. A genome-wide association study on cannabis use and BD has identified shared genetic associations between CUD and BD(Pasman et al., 2018). Significant genetic associations have also been identified between chronotype and psychiatric traits, including depression symptoms(Jones et al., 2019) and lifetime cannabis use(Winiger et al., 2021), suggesting that circadian rhythm disturbances may indeed be related to both conditions.

As summarized, current literature supports two competing models of BD and cannabis use: 1) cannabis use directly contributes to the worsening of BD symptoms; and 2) a shared underlying factor predisposes individuals towards both BD and CUD. As described above, a compromised circadian system may be a predisposing factor for the latter model of BD and cannabis use, thus in this study, we aimed to further investigate the latter model 1) determine whether CUD was associated with BD symptom onset, 2) determine whether cannabis use initiation relative to BD symptom onset impacts BD symptom onset, and 3) determine whether chronotype, as a proxy for circadian rhythm disturbance, was associated with BD symptom onset, and whether co-morbid CUD moderated this relationship. Here, we used data collected from patients with BD type I, who participated in the Pharmacogenomics of Bipolar Disorder (PGBD) study that either had a diagnosed co-morbid CUD or did not. We hypothesized the following: 1) CUD would be associated with an earlier age of mood symptom onset relative to those with no CUD, 2) greater eveningness would be associated with earlier BD symptom onset and CUD would not moderate this relationship, and 3) initiation of cannabis use prior to mood symptom onset would not be associated with earlier age of BD symptom onset. If supported these hypotheses would help disentangle the two competing models and provide further support that CUD and BD share an underlying mechanism.

## 2. Methods

### 2.1. Participants

Participant data were derived from the PGBD study dataset. The Diagnostic Interview for Genetic Studies (DIGS) (Oedegaard et al., 2016) was used to assess psychiatric and substance use disorders. BD diagnosis criteria were defined using the Diagnostic and Statistical Manual of Mental Disorders IV. Only participants with BD I (n = 212) were analyzed for this study. The DIGS and other medical records were used to give as accurate descriptions of psychiatric symptoms and substance use as possible.

### 2.2. Bipolar disorder symptom variables

The Final Best Estimate method(Nurnberger et al., 1994) was used to determine mood symptom age of onset (AAO), lifetime number of mood episodes and chronicity of cannabis use. Depression and mania episode density (ED) was calculated for each participant, which measures the number of mood episodes in a given amount of time, a method described by Strejilevich et al.(Strejilevich et al., 2019, 2024). ED can be used as a proxy for lifetime burden of mood episodes, where higher ED corresponds to greater lifetime burden of mood episodes. Here, we use ED to account for differences in participant age. All BD symptom variables are listed and defined in Table 1.

### 2.3. Cannabis use disorder

Participants were stratified into two groups: those with diagnosed cannabis abuse or dependence (CUD+; n = 53) and those with no cannabis abuse or dependence (CUD−; n = 159). For CUD patients with cannabis use age of onset data available, cannabis use onset relative to mania or depression symptom onset was dichotomized as those who initiated cannabis use prior to or within the same year of mood symptom onset (Pre-onset) and those who initiated cannabis use after mood symptom onset (Post-onset). Pre- and post-onset groups were calculated relative to mania and depression onset separately.

### 2.4. Chronotype

Chronotype was measured in units of morningness. Participants were surveyed for chronotype using the Basic Language Morningness Scale (BALM)(Brown, 1993; Smith et al., 1989). The BALM is a 13-item composite self-report scale used to measure circadian rhythms. Summary scores consisted of the sum of all 13 items, with higher summary scores indicating a higher level of morningness.

### 2.5. Statistical analysis

Assumptions for equal variances (Levene’s or Box’s test of equality) and normality (Shapiro-Wilks test) were tested for all variables; variance and normality were tested across the entire sample and within each group. Non-parametric testing was used throughout to account for non-normal distributions, unequal variances and unequal sample sizes. Correlations between BALM score and BD mood symptoms were tested using Spearman’s correlations. Differences (i.e., age, mood symptoms and BALM score) between CUD and CUD-groups were analyzed using Mann-Whitney U tests. Chi-square testing was used to determine group differences for nominal variables (i.e., sex, psychosis symptoms and suicidality). Group differences between no CUD, cannabis use pre-symptom onset and cannabis use post-symptom onset was first analyzed using Kruskal-Wallis tests, followed by pairwise Mann-Whitney U tests. The α level was set at 0.05 and Bonferroni multiple testing correction threshold was used (p < 0.0167). A moderated linear regression was performed using the SPSS package PROCESS (Hayes, 2022) to test whether CUD moderates the relationship between chronotype and BD symptoms. Due to the non-normal distribution of BD symptom variables, log transformation was used to achieve normality. As a sensitivity analysis, regression analyses were conducted with both non-transformed and transformed data; results were consistent thus the findings are presented using non-transformed variables BALM summary score was used as the independent variable, BD symptoms as the dependent variables, CUD as a moderating variable^28^. All statistical analyses were performed using SPSS 28.0 (Chicago, IL, USA).

## 3. Results

### 3.1. BD with comorbid CUD was associated with earlier onset of mood symptoms and affective psychosis

Patients with CUD were significantly younger than patients without CUD. There was a smaller proportion of females in the CUD group compared to the CUD-group (Table 2), however there were no significant group differences between males and females on any of the BD symptom variables or BALM score. A greater proportion of CUD patients reported having psychotic symptoms in at least 2 episodes compared to patients with no CUD (Х^2^ = 8.86, p < 0.05; Table 2). Significantly greater percentage of CUD-patients reported never having suicidal thoughts compared to patients with CUD. (Х^2^ = 4.75, p < 0.05, Table 2). Consistent with previous literature, CUD + patients had significantly lower morningness as measured by BALM compared to BD patients without CUD (U = 3298, p < 0.05, Table 2). There were no significant differences in depression ED between CUD- and CUD groups. However, mania ED was significantly higher in CUD patients (U = 3236, p < 0.05, Table 3) and mania AOO was significantly earlier in CUD patients (U = 2580, p < 0.001, Table 3), compared to CUD-patients. Depression AOO was earlier in CUD patients compared to CUD-patients but the difference did not reach statistical significance (U = 3433, p = 0.086, Table 3).

### 3.2. Chronotype was associated with mood symptom age of onset

**Fig. 1.**
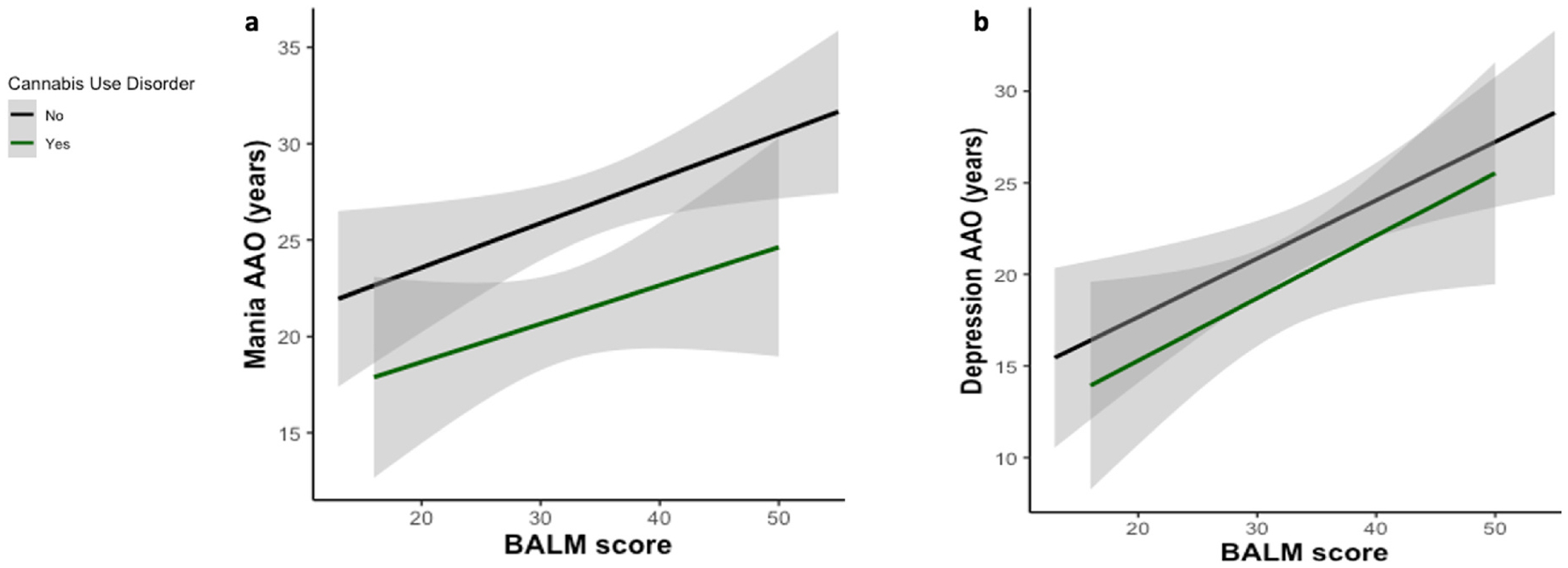
Simple slopes for the association between Basic Language Morningness Scale (BALM) score and mood symptom age of onset (AAO) in patients with and without a diagnosed cannabis use disorder (CUD). a) BALM score was significantly associated with mania AAO (b = 0.23, t = 2.48, p = 0.01), though CUD did not significantly moderate this relationship. b) BALM score was significantly associated with depression AAO (b = 0.32, t = 3.19, p = 0.002), though CUD did not significantly moderate this association. Shaded areas indicate 95% confidence intervals.

We then tested whether CUD moderates the relationship between chronotype and BD symptom onset/ED. BALM scores were significantly associated with mania AAO and depression AAO. Greater morningness (higher BALM scores) was associated with a later AAO for mania (b = 0.23, t = 2.48, p = 0.01, Fig. 1a) and depression (b = 0.32, t = 3.19, p < 0.01, Fig. 1b). Despite the significant difference between mood symptom AAO between CUD and CUD-patients, CUD did not significantly moderate the relationship between chronotype and mood symptom AAO (Table 4). Cannabis use AAO relative to mood symptom AAO did not significantly moderate the relationship between chronotype and mood symptom AAO.

### 3.3. Cannabis use onset prior to mood symptom onset was significantly associated with later mood symptom age of onset

**Fig. 2.**
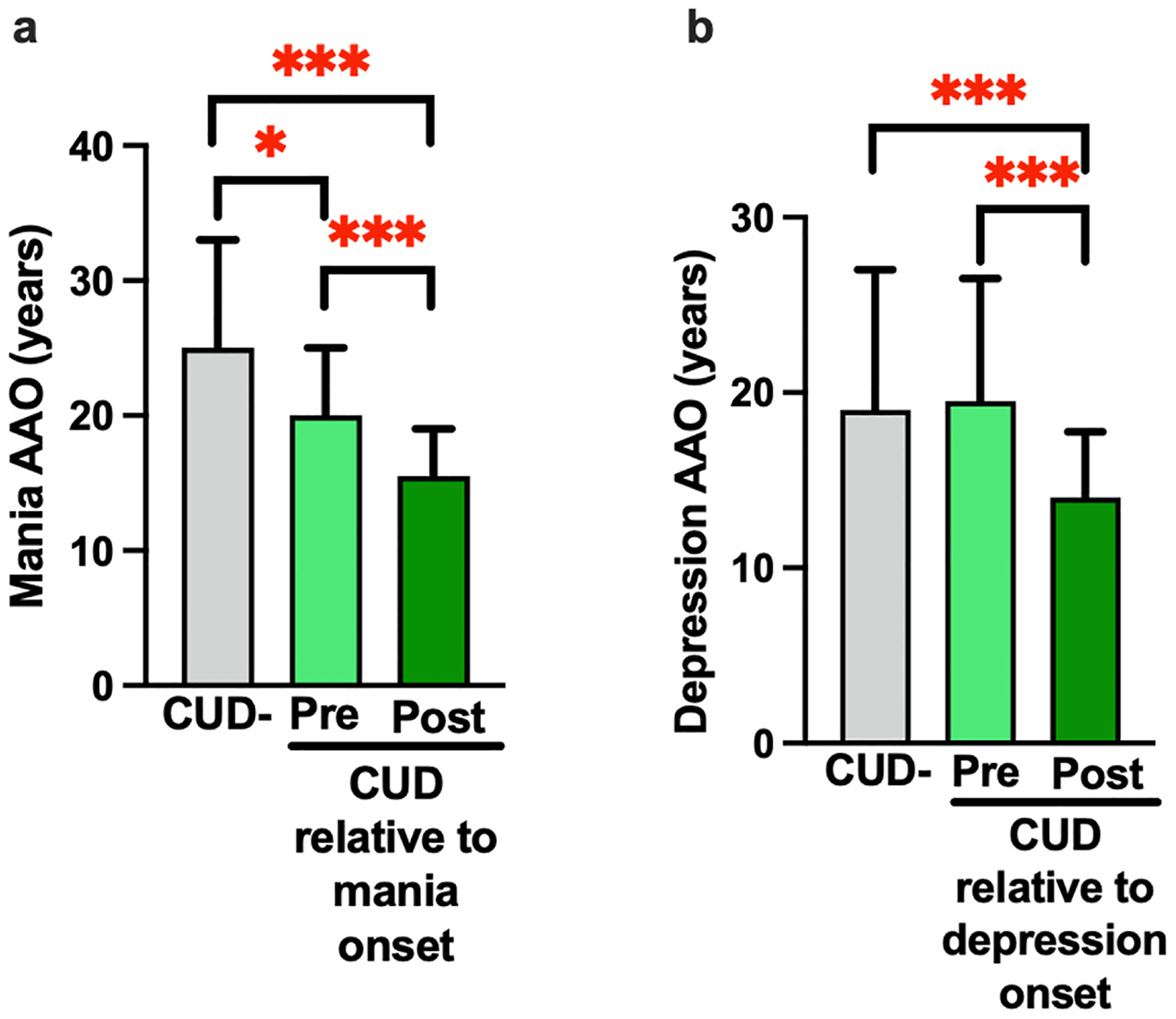
Cannabis use onset pre-BD symptom age of onset (AAO) is associated with a later mood symptom AAO relative to cannabis use post-BD symptom AAO. a) Mania AAO was significantly earlier in the post-mania onset group (n = 14) compared to both the pre-onset group (n = 39); U = 105, p < 0.001) and CUD-group (U = 311.5, p < 0.001). Mania AAO was also significantly later in CUD-compared to pre-onset participants (U, 2268.5, p = 0.012). b) Post depression onset cannabis use (n = 24) was associated with significantly earlier depression AAO compared to CUD- (U = 988, p < 0.001) and pre-depression onset cannabis use (n = 28; U = 121, p < 0.001). Data represents median and interquartile range; *p < 0.05, ***p < 0.001.

There was a significant difference in mania AAO between no CUD, cannabis use pre (n = 39) and post symptom onset (n = 14) groups (H = 24.73, p < 0.001, Fig. 2a). Mania AAO was significantly later in CUD-patients, compared to those who initiated cannabis use both pre (U = 2268.5, p = 0.01). and post-mania onset (U = 311.5, p < 0.001). People who initiated cannabis use post-symptom onset also had significantly earlier mania AAO compared to those who initiated cannabis use pre-symptom onset (U = 105, p < 0.001). Depression AAO was significantly different between cannabis use onset groups (H = 16.92, p < 0.001, Fig. 2b). Depression AAO was later in patients with no CUD (U = 988, p < 0.001) and in patients that reported initiating cannabis use pre-depression onset (n = 28; U = 121, p < 0.001) compared to patients who initiated cannabis use post-depression onset (n = 24). Initiation of cannabis use relative to symptom onset was not associated with depression or mania ED.

## 4. Discussion

Our data support previous reports that CUD in BD is associated with an earlier onset, greater severity of symptoms and worse clinical features. Specifically, we found that people with BD and comorbid CUD had an earlier AAO for mania and depression, greater number of mania episodes per year and more often reported presence of affective psychosis. Our data indicate that while both chronotype and CUD were independently associated with mood symptom AAO, there was no interactive effect of CUD and chronotype on mood symptom onset. Importantly, we found that cannabis use onset prior to mood symptom onset was not associated with earlier emergence of mood symptoms; rather, mood symptom AAO was *later* in BD patients who reported initiating cannabis use prior to mood symptom onset, compared to BD patients who reporting initiating cannabis use after mood symptom onset. These findings help to clarify the temporal relationship between cannabis use and mood symptom onset, a critical step towards elucidating the causal relationship between these two psychiatric conditions.

Previous studies have similarly investigated the temporal relationship between substance dependence and BD. For example, Martínez-Ortega et al. assessed nicotine dependence onset relative to BD onset in a large cohort. The authors reported significant differences in lifetime BD symptoms and comorbid conditions between patients that reported nicotine dependence prior to BD onset (*n* = 135) compared to patients who reported nicotine dependence following BD onset (*n* = 386) (Martínez-Ortega et al., 2013). The authors also analyzed nicotine dependence relative to major depression onset using similar methods (Martínez-Ortega et al., 2017). These studies have added meaningful knowledge on the temporal relationship between mood disorders and substance use thus, we employed a similar approach by comparing cannabis use onset relative to both mania and depression onset in patients with BD. Our findings are consistent with previous literature in which CUD was associated with an earlier age of mood symptom onset and psychosis in BD(De Hert et al., 2011; Kuhns et al., 2021). For example, BD/CUD patients have been reported to have the lowest age of BD onset (mean age of cannabis use onset = 18.6 years) compared to BD patients who did not use cannabis and patients who engaged in cannabis use but did not meet diagnostic criteria for CUD(Lagerberg et al., 2014). Excessive cannabis use has also been found to predict first incidence mood episodes in BD after controlling for a number of potentially confounding factors (i.e., sociodemographic factors, childhood trauma, family psychiatric history, other substance use disorders and other mental disorders)(Cougle et al., 2015; Van Laar et al., 2007). Initiation of cannabis use pre- or post-symptom onset were both associated with an earlier age of mood symptom onset(Lagerberg et al., 2011). Our results are in part consistent with this report, such that initiation of cannabis use both pre- and post-onset of mania symptoms was associated with an early age of BD onset compared to BD patients without CUD. Our data further expand on these previous reports and demonstrate that initiation of cannabis use pre-mania onset is associated with a significantly later mania AAO compared to those initiating cannabis use post-mania onset. Further, depression AAO was statistically similar between the CUD-patients and the patients that reported initiating cannabis use pre-depression onset. These findings further strengthen the hypothesis that while CUD is associated with earlier age of onset of BD symptoms, initiation of cannabis use is not directly a major driver of early BD onset.

Our data on cannabis use onset relative to mood symptom onset indicate that other factors may contribute to BD symptom onset and severity and also predispose people towards initiation of problematic cannabis use and development of CUD. As described previously, circadian rhythm disruption could be one such factor that impacts both BD symptom onset and problematic cannabis use. A recent review indicated that individuals with circadian rhythm disturbances have a significantly elevated risk for BD(Scott et al., 2022). Another study supports these findings by demonstrating that higher eveningness in adolescents and young adults significantly predicted next-year depressive symptoms(Sasser et al., 2023). A longitudinal analysis of substance use in young adults and adolescents found that greater eveningness significantly predicted next-year cannabis use(Hasler et al., 2022). Lastly, mendelian randomization analyses have provided evidence that lower genetic loading for morningness significantly increases the liability of depression symptoms(Jones et al., 2019), whereas increased insomnia increases the risk of cannabis use initiation(Winiger et al., 2021). Here, we demonstrated a significant difference in chronotype between BD patients with and without co-morbid CUD. While greater morningness was associated with later mood symptom AAO, CUD did not moderate the relationship between chronotype and mood symptom AAO. The lack of interaction between CUD and chronotype on BD symptom onset indicates that CUD does not directly impact BD symptoms. These data, combined with existing literature supports the model that CUD and BD are distinct conditions similarly impacted by circadian rhythm disruption.

The results presented here add new evidence on the interrelationship between CUD, BD and circadian rhythms, however this study has several limitations. Although, participant data were clearly defined and comprehensively validated through clinical interviews and reviews, our analyses did not include current depression and mania symptom data. Importantly, we have previously reported that chronotype also predicts current depressive and mania symptoms in this cohort(McCarthy et al., 2018); thus, future analyses may investigate potential interactions between CUD and chronotype on current mood symptoms. It is also critical to note that there was no limiting eligibility criteria for mood state for this study, as such while many participants were euthymic, some presented with depression and mania/hypomania that could have impacted their memory. This recall bias may affect the participants’ ability to accurately recall number of mood episodes, which may have led to an overestimation or underestimation of significance in the relationships among episode density, chronotype and CUD. Additionally, our dataset only included information on current cannabis abuse or dependence diagnoses but did not include whether participants engage in regular cannabis use that does not reach the threshold of a cannabis abuse or dependence diagnosis. This distinction is important to make for future studies considering many people with BD report regular cannabis for symptom management. In other populations, regular cannabis use has been shown to reduce symptom severity, in a dose-dependent manner, such that lighter use improves symptoms whereas heavy cannabis use worsens symptoms (Ellingson et al., 2021; Kallianpur et al., 2020; Thames et al., 2016). Thus, future studies evaluating the effects of cannabis use frequency on mood symptoms could yield important insight into manageable cannabis use practices in a population where cannabis use is highly prevalent. Multiple testing, unequal sample sizes and smaller sample sizes may have affected the statistical power of our analyses, particularly in the cannabis use relative to mania onset groups. Additionally, the significant association between earlier age of mania onset in participants and initiating cannabis use before mania onset could also indicate that some people with BD initiate cannabis use to self-medicate prodromal symptoms before full illness onset. As described previously, there is strong supporting evidence of a shared underlying predisposition for BD and CUD. However, future studies monitoring the emergence of cannabis use and subclinical symptoms in adolescents and young adults at-risk for developing BD could further disentangle these two models of BD and cannabis use.

Here, we propose that circadian rhythm disruption contributes to both CUD and BD. Future studies could evaluate whether chronotype could be used as a biomarker to predict mood symptom onset in young adults at risk for developing BD or identify patient groups at greater risk for developing CUD. Our study is consistent with others indicating that circadian rhythm disruption could be a promising target for identifying CUD and BD risk. More research should be conducted to better understand the underlying neurobiology of this relationship, and whether circadian rhythms can be leveraged to improve BD and CUD outcomes.

## Figures and Tables

**Table 1:** BD symptom variables and definitions.

BD symptom variables	Definition
Mania age of onset (mania AOO)	Age at first diagnosed mania episode
Depression age of onset (depression AOO)	Age at first diagnosed depression episode
Mania episode density	Lifetime total number of mania episodes Age - mania age of onset
Depression episode density	Lifetime total number of depression episodes Age – depression age of onset
Affective Psychosis	Presence of psychosis during mood episodes:
	No (0–1 episodes of psychosis)
	Yes (2+ episodes with psychosis)
Suicidality	Presence of suicidality:
	Never/none
	Thoughts of suicide
	Suicide attempts

**Table 2:** Demographic and clinical data on patients with bipolar disorder (BD), with and without comorbid cannabis use disorder (CUD). Data reported are mean (standard deviation) and percent of group.

	CUD-n = 159	CUD n = 53	Group differences
**Age**	45.6(14.3)	37.5(12.5)	CUD- > CUD
**Sex *F/M***	59%/41%	38%/62%	p < 0.05
**Affective psychosis**			
*0–1 episodes*	64%	34%	CUD- > CUD
*2+ episodes*	36%	66%	CUD- < CUD
**Suicidality**			
*Never*	30%	16%	CUD- > CUD
*Thoughts*	35%	42%	ns
*Attempted*	35%	42%	ns
Basic Language Morningness Scale (BALM) score	36 (11)	32 (12)	CUD- > CUD

**Table 3:** Mood symptom age of onset and episode density in patients with bipolar disorder (BD), with and without comorbid cannabis use disorder (CUD). Data reported are median (Interquartile range).

	CUD-n = 159	CUD n = 53	U	p	Group differences
Mania episode density	0.32 (0.12–0.59)	0.36 (0.19–0.98)	3236	0.021	CUD- < CUD
Depression episode density	0.33 (0.17–0.79)	0.39 (0.17–0.99)	3711	0.47	ns
Mania age of onset	25 (19–33)	19 (16–22)	2580	<0.001	CUD- > CUD
Depression age of onset	19 (15–27)	18 (13–22)	3433	0.086	ns

**Table 4:** Summaries for linear regression models of mania and depression age of onset (AAO).

Mania AAO				
	*b*	SE	*t*	*p*
BALM score	0.32	0.10	3.13	0.002
CUD status	−2.84	7.15	−0.40	0.69
BALM score x CUD status	0.02	0.21	0.11	0.91
Depression AAO				
	*b*	SE	*t*	*p*
BALM score	0.23	0.09	2.48	0.01
CUD status	−4.25	6.62	−0.64	0.52
BALM score x CUD status	−0.03	0.20	−0.17	0.87

## References

[R1] Agrawal A, Nurnberger JI, Lynskey MT, 2011. Cannabis involvement in individuals with bipolar disorder. Psychiatr. Res 185, 459. 10.1016/J.PSYCHRES.2010.07.007. PMC2976789 20674039

[R2] Babson KA, Sottile J, Morabito D, 2017. Cannabis, cannabinoids, and sleep: a review of the literature. Curr. Psychiatr. Rep 10.1007/s11920-017-0775-9. 28349316

[R3] Barrigón ML, Gurpegui M, Ruiz-Veguilla M, Diaz FJ, Anguita M, Sarramea F, Cervilla J, 2010. Temporal relationship of first-episode non-affective psychosis with cannabis use: a clinical verification of an epidemiological hypothesis. J. Psychiatr. Res 44, 413–420. 10.1016/J.JPSYCHIRES.2009.10.004. 19900684

[R4] Berdynaj D, Boudissa SN, Grieg MS, Hope C, Mahamed SH, Norbury R, 2016. Effect of chronotype on emotional processing and risk taking. Chronobiol. Int 33, 406–418. 10.3109/07420528.2016.1146739. 27030174

[R5] Brown FM, 1993. Psychometric equivalence of an improved Basic Language Morningness (BALM) scale using industrial population within comparisons. Ergonomics 36, 191–197. 10.1080/00140139308967872. 8440216

[R6] Cougle JR, Hakes JK, Macatee RJ, Chavarria J, Zvolensky MJ, 2015. Quality of life and risk of psychiatric disorders among regular users of alcohol, nicotine, and cannabis: an analysis of the National Epidemiological Survey on Alcohol and Related Conditions (NESARC). J. Psychiatr. Res 66–67, 135–141. 10.1016/J.JPSYCHIRES.2015.05.004. 26022838

[R7] De Hert M, Wampers M, Jendricko T, Franic T, Vidovic D, De Vriendt N, Sweers K, Peuskens J, van Winkel R, 2011. Effects of cannabis use on age at onset in schizophrenia and bipolar disorder. Schizophr. Res 126, 270–276. 10.1016/J.SCHRES.2010.07.003. 20674280

[R8] Ellingson JM, Hinckley JD, Ross JM, Schacht JP, Bidwell LC, Bryan AD, Hopfer CJ, Riggs P, Hutchison KE, 2021. The neurocognitive effects of cannabis across the lifespan. Curr Behav Neurosci Rep 8, 124–133. 10.1007/S40473-021-00244-7. 35979200 PMC9377647

[R9] Gold AK, Otto MW, Deckersbach T, Sylvia LG, Nierenberg AA, Kinrys G, 2018. Substance use comorbidity in bipolar disorder: a qualitative review of treatment strategies and outcomes. Am. J. Addict 27, 188–201. 10.1111/AJAD.12713. 29596721

[R10] Gold AK, Sylvia LG, 2016. The role of sleep in bipolar disorder. Nat. Sci. Sleep 10.2147/NSS.S85754. PMC4935164 27418862

[R11] Harvey AG, Talbot LS, Gershon A, 2009. Sleep disturbance in bipolar disorder across the lifespan. Clin. Psychol. Sci. Pract 16, 256–277. 10.1111/j.1468-2850.2009.01164.x. PMC3321357 22493520

[R12] Hasler BP, Graves JL, Wallace ML, Claudatos S, Franzen PL, Nooner KB, Brown SA, Tapert SF, Baker FC, Clark DB, 2022. Self-reported sleep and circadian characteristics predict alcohol and cannabis use: a longitudinal analysis of the National Consortium on Alcohol and Neurodevelopment in Adolescence Study. Alcohol Clin. Exp. Res 46, 848–860. 10.1111/ACER.14808. 35579668 PMC9179040

[R13] Hasler BP, Soehner AM, Clark DB, 2014. Circadian rhythms and risk for substance use disorders in adolescence. Curr. Opin. Psychiatr 27, 460. 10.1097/YCO.0000000000000107. PMC4227308 25247459

[R14] Hayes AF, 2022. Introduction to Mediation, Moderation, and Conditional Process Analysis: A Regression-Based Approach, third ed. Guilford Press, NewYork.

[R15] Jones SE, Lane JM, Wood AR, van Hees VT, Tyrrell J, Beaumont RN, Jeffries AR, Dashti HS, Hillsdon M, Ruth KS, Tuke MA, Yaghootkar H, Sharp SA, Jie Y, Thompson WD, Harrison JW, Dawes A, Byrne EM, Tiemeier H, Allebrandt KV, Bowden J, Ray DW, Freathy RM, Murray A, Mazzotti DR, Gehrman PR, Lawlor DA, Frayling TM, Rutter MK, Hinds DA, Saxena R, Weedon MN, 2019. Genome-wide association analyses of chronotype in 697,828 individuals provides insights into circadian rhythms. Nat. Commun 10 (1), 1–11. 10.1038/s41467-018-08259-7, 2019 10. 30696823 PMC6351539

[R16] Kallianpur KJ, Birn R, Ndhlovu LC, Souza SA, Mitchell B, Paul R, Chow DC, Kohorn L, Shikuma CM, 2020. Impact of cannabis use on brain structure and function in suppressed HIV infection. J. Behav. Brain Sci 10, 344. 10.4236/jbbs.2020.108022. 32968547 PMC7508465

[R17] Kuhns L, Kroon E, Colyer-Patel K, Cousijn J, 2021. Associations between cannabis use, cannabis use disorder, and mood disorders: longitudinal, genetic, and neurocognitive evidence. Psychopharmacology 239 (5), 1231–1249. 10.1007/S00213-021-06001-8, 2021 239. 34741634 PMC9520129

[R18] Lagerberg TV, Kvitland LR, Aminoff SR, Aas M, Ringen PA, Andreassen OA, Melle I, 2014. Indications of a dose–response relationship between cannabis use and age at onset in bipolar disorder. Psychiatr. Res 215, 101–104. 10.1016/J.PSYCHRES.2013.10.029. 24262665

[R19] Lagerberg TV, Sundet K, Aminoff SR, Berg AO, Ringen PA, Andreassen OA, Melle I, 2011. Excessive cannabis use is associated with earlier age at onset in bipolar disorder. Eur. Arch. Psychiatr. Clin. Neurosci 261, 397–405. 10.1007/S00406-011-0188-4. PMC3159738 21267743

[R20] Martínez-Ortega JM, Franco S, Rodríguez-Fernández JM, Gutíerrez-Rojas L, Wang S, Gurpegui M, 2017. Temporal sequencing of nicotine dependence and major depressive disorder: a U.S. national study. Psychiatr. Res 250, 264–269. 10.1016/J.PSYCHRES.2017.01.087. 28183022

[R21] Martínez-Ortega JM, Goldstein BI, Gutíerrez-Rojas L, Sala R, Wang S, Blanco C, 2013. Temporal sequencing of nicotine dependence and bipolar disorder in the national epidemiologic survey on alcohol and related conditions (NESARC). J. Psychiatr. Res 47, 858–864. 10.1016/J.JPSYCHIRES.2013.03.012. 23582710 PMC3674324

[R22] McCarthy MJ, Wei H, Nievergelt CM, Stautland A, Maihofer AX, Welsh DK, Shilling P, Alda M, Alliey-Rodriguez N, Anand A, Andreasson OA, Balaraman Y, Berrettini WH, Bertram H, Brennand KJ, Calabrese JR, Calkin CV, Claasen A, Conroy C, Coryell WH, Craig DW, D’Arcangelo N, Demodena A, Djurovic S, Feeder S, Fisher C, Frazier N, Frye MA, Gage FH, Gao K, Garnham J, Gershon ES, Glazer K, Goes F, Goto T, Harrington G, Jakobsen P, Kamali M, Karberg E, Kelly M, Leckband SG, Lohoff F, McInnis MG, Mondimore F, Morken G, Nurnberger JI, Obral S, Oedegaard KJ, Ortiz A, Ritchey M, Ryan K, Schinagle M, Schoeyen H, Schwebel C, Shaw M, Shekhtman T, Slaney C, Stapp E, Szelinger S, Tarwater B, Zandi PP, Kelsoe JR, 2018. Chronotype and cellular circadian rhythms predict the clinical response to lithium maintenance treatment in patients with bipolar disorder. Neuropsychopharmacology 44 (3), 620–628. 10.1038/s41386-018-0273-8, 2018 44. 30487653 PMC6333516

[R23] Menculini G, Steardo LJ, Verdolini N, D’Angelo M, Chipi E, Cirimbilli F, Orsolini L, Volpe U, De Fazio P, Tortorella A, 2023. Chronotype is associated with affective temperaments, clinical severity and worse treatment outcomes in bipolar disorders: results from a two-center, cross-sectional study. Int. J. Psychiatr. Clin. Pract 10.1080/13651501.2022.2160763. 36622183

[R24] Miranda A, Holloway B, Peek E, Young JW, Perry W, Minassian A, 2023. Cannabis use patterns and their effects on risky decision-making in bipolar disorder. Society of Biological Psychiatry S174. S174.

[R25] Monti JM, Pandi-Perumal SR, 2022. Clinical management of sleep and sleep disorders with cannabis and cannabinoids. Clin Neuropharmacol Publish Ah 1–5. 10.1097/wnf.0000000000000494. 35221321

[R26] Nurnberger JI, Blehar MC, Kaufmann CA, York-Cooler C, Simpson SG, Harkavy-Friedman J, Severe JB, Malaspina D, Reich T, 1994. Diagnostic interview for genetic studies: rationale, unique features, and training. Arch. Gen. Psychiatr 51, 849–859. 10.1001/ARCHPSYC.1994.03950110009002. 7944874

[R27] Oedegaard KJ, Alda M, Anand A, Andreassen OA, Balaraman Y, Berrettini WH, Bhattacharjee A, Brennand KJ, Burdick KE, Calabrese JR, Calkin CV, Claasen A, Coryell WH, Craig D, DeModena A, Frye M, Gage FH, Gao K, Garnham J, Gershon E, Jakobsen P, Leckband SG, McCarthy MJ, McInnis MG, Maihofer AX, Mertens J, Morken G, Nievergelt CM, Nurnberger J, Pham S, Schoeyen H, Shekhtman T, Shilling PD, Szelinger S, Tarwater B, Yao J, Zandi PP, Kelsoe JR, 2016. The Pharmacogenomics of Bipolar Disorder study (PGBD): identification of genes for lithium response in a prospective sample. BMC Psychiatr. 16. 10.1186/S12888-016-0732-X. PMC4857276 27150464

[R28] Pasman JA, Verweij KJH, Gerring Z, Stringer S, Sanchez-Roige S, Treur JL, Abdellaoui A, Nivard MG, Baselmans BML, Ong JS, Ip HF, van der Zee MD, Bartels M, Day FR, Fontanillas P, Elson SL, de Wit H, Davis LK, MacKillop J, Derringer JL, Branje SJT, Hartman CA, Heath AC, van Lier PAC, Madden PAF, Mägi R, Meeus W, Montgomery GW, Oldehinkel AJ, Pausova Z, Ramos-Quiroga JA, Paus T, Ribases M, Kaprio J, Boks MPM, Bell JT, Spector TD, Gelernter J, Boomsma DI, Martin NG, MacGregor S, Perry JRB, Palmer AA, Posthuma D, Munafò MR, Gillespie NA, Derks EM, Vink JM, 2018. GWAS of lifetime cannabis use reveals new risk loci, genetic overlap with psychiatric traits, and a causal influence of schizophrenia. Nat. Neurosci 21, 1161–1170. 10.1038/S41593-018-0206-1. 30150663 PMC6386176

[R29] Pinto JV, Medeiros LS, Santana da Rosa G, Santana de Oliveira CE, Crippa JA de S, Passos IC, Kauer-Sant’Anna M, 2019. The prevalence and clinical correlates of cannabis use and cannabis use disorder among patients with bipolar disorder: a systematic review with meta-analysis and meta-regression. Neurosci. Biobehav. Rev 101, 78–84. 10.1016/J.NEUBIOREV.2019.04.004. 30974123

[R30] Santos IM, Bem-Haja P, Silva A, Rosa C, Queiroz DF, Alves MF, Barroso T, Cerri L, Silva CF, 2023. The interplay between chronotype and emotion regulation in the recognition of facial expressions of emotion. Behav. Sci 13. 10.3390/BS13010038. PMC9855169 36661610

[R31] Sasser J, Waddell JT, Doane LD, 2023. If you (Don’t) Snooze, Do You Use? Prospective Links Between Adolescent Sleep Patterns and Substance Use and Depression. Int. J. Ment. Health Addiction 1–18. 10.1007/S11469-023-01027-9/TABLES/4.

[R32] Scott J, Etain B, Miklowitz D, Crouse JJ, Carpenter J, Marwaha S, Smith D, Merikangas K, Hickie I, 2022. A systematic review and meta-analysis of sleep and circadian rhythms disturbances in individuals at high-risk of developing or with early onset of bipolar disorders. Neurosci. Biobehav. Rev 135. 10.1016/J.NEUBIOREV.2022.104585. PMC8957543 35182537

[R33] Serrano-Serrano AB, Marquez-Arrico JE, Navarro JF, Martinez-Nicolas A, Adan A, 2021. Circadian Characteristics in Patients under Treatment for Substance Use Disorders and Severe Mental Illness (Schizophrenia, Major Depression and Bipolar Disorder). J. Clin. Med 10. 10.3390/JCM10194388. PMC8509477 34640406

[R34] Sideli L, Quigley H, La Cascia C, Murray RM, 2019. Cannabis Use and the Risk for Psychosis and Affective Disorders. J. Dual Diagn 16, 22–42. 10.1080/15504263.2019.1674991. 31647377

[R35] Smith CS, Reilly C, Midkiff K, 1989. Evaluation of three circadian rhythm questionnaires with suggestions for an improved measure of morningness. J. Appl. Psychol 74, 728–738. 10.1037/0021-9010.74.5.728. 2793773

[R36] Strejilevich S, Samamé C, Marengo E, Godoy A, Smith J, Camino S, Oppel M, Sobrero M, López Escalona L, 2024. Can we predict a “tsunami”? Symptomatic and syndromal density, mood instability and treatment intensity in people with bipolar disorders under a strict and long lockdown. J. Affect. Disord 351. 10.1016/j.jad.2024.02.007. 38341152

[R37] Strejilevich Sergio, Szmulewicz A, Igoa A, Marengo E, Caravotta P, Martino D, Strejilevich S, Program BD, 2019. Episodic density, subsyndromic symptoms, and mood instability in late-life bipolar disorders: A 5-year follow-up study. Int. J. Geriatr. Psychiatr 10.1002/gps.5094. 30864181

[R38] Takaesu Y, Inoue Y, Ono K, Murakoshi A, Futenma K, Komada Y, Inoue T, 2018. Circadian Rhythm Sleep-Wake Disorders Predict Shorter Time to Relapse of Mood Episodes in Euthymic Patients With Bipolar Disorder: A Prospective 48-Week Study. J. Clin. Psychiatry 79. 10.4088/JCP.17M11565. 29286593

[R39] Tamura EK, Oliveira-Silva KS, Ferreira-Moraes FA, Marinho EAV, Guerrero-Vargas NN, 2021. Circadian rhythms and substance use disorders: A bidirectional relationship. Pharmacol. Biochem. Behav 201, 173105. 10.1016/J.PBB.2021.173105. 33444601

[R40] Thames AD, Mahmood Z, Burggren AC, Karimian A, Kuhn TP, 2016. Combined effects of HIV and marijuana use on neurocognitive functioning and immune status. AIDS Care 28, 628–632. 10.1080/09540121.2015.1124983. 26694807 PMC4950932

[R41] Van Laar M, Van Dorsselaer S, Monshouwer K, De Graaf R, 2007. Does cannabis use predict the first incidence of mood and anxiety disorders in the adult population? Addiction 102, 1251–1260. 10.1111/J.1360-0443.2007.01875.X. 17624975

[R42] Veen ND, Selten JP, Van Der Tweel I, Feller WG, Hoek HW, Kahn RS, 2004. Cannabis Use and Age at Onset of Schizophrenia. Am. J. Psychiatr 161, 501–506. 10.1176/APPI.AJP.161.3.501/ASSET/IMAGES/M616F2.JPEG. 14992976

[R43] Winiger EA, Ellingson JM, Morrison CL, Corley RP, Pasman JA, Wall TL, Hopfer CJ, Hewitt JK, 2021. Sleep deficits and cannabis use behaviors: an analysis of shared genetics using linkage disequilibrium score regression and polygenic risk prediction. Sleep 44. 10.1093/SLEEP/ZSAA188. PMC7953210 32935850

